# CREATION OF A COMMON METRIC OF HEALTH STATUS IN THE HARMONIZED DATASET OF THE ATHLOS PROJECT

**DOI:** 10.1093/geroni/igz038.2941

**Published:** 2019-11-08

**Authors:** Albert Sanchez-Niubo, Francisco Felix Caballero, Christina Daskalopoulou, Javier de la Fuente, Alejandro de la Torre, Iago Giné Vazquez, Yu-Tzu Wu, Matthew Prina

**Affiliations:** 1 Parc Sanitari Sant Joan de Déu, Universitat de Barcelona, Barcelona, Spain; 2 Centro de Investigación Biomédica en Red de Salud Mental, CIBERSAM, Madrid, Madrid, Spain; 3 Social Epidemiology Research Group. Health Service and Population Research Department, Institute of Psychiatry, Psychology & Neuroscience, King’s College London, London, England, United Kingdom; 4 Department Preventive Medicine and Public Health, Universidad Autónoma de Madrid, Madrid, Madrid, Spain; 5 Parc Sanitari Sant Joan de Déu, Baecelona, Catalonia, Spain

## Abstract

Although life longevity has increased across the world, evidence suggests some heterogeneity of the ageing process across individuals. To investigate different ageing patterns, the ATHLOS project harmonised data from 411,000 individuals across 17 existing cohort studies. The harmonised dataset provides comparable information on functioning measures, cognition, mental health, sociodemographic and lifestyle behaviours. To measure the process of healthy ageing across time and cohorts, we employed a Bayesian Multilevel Item Response Theory(IRT) and created a common metric of health status by using items of functioning. The IRT measurement model includes parameters describing the difficulty and discriminatory power of each item. We adopted the Bayesian Multilevel framework as it allows item parameters to vary among studies and the simultaneous estimation of all parameters under a Markov Chain Monte Carlo(MCMC) method. Finally, we assessed the predictive validity of the metric against mortality by performing a Receiver Operating Characteristic(ROC) curve analysis.

